# Adhesion Study of *Pseudomonas aeruginosa* on Different Urinary Catheter Materials

**DOI:** 10.4314/ejhs.v35i2.10

**Published:** 2025-03

**Authors:** Yogesh B Bele, Prashant V Thakare, Niraj A Ghanwate

**Affiliations:** 1 Department of Microbiology, Sant Gadge Baba Amravati University, Amravati, Maharashtra, 444602, India; 2 Department of Biotechnology, Sant Gadge Baba Amravati University, Amravati, Maharashtra, 444602, India

**Keywords:** Adherence, biofilm, catheter materials, Pseudomonas aeruginosa, scanning electron microscopy

## Abstract

**Background:**

Foley catheters, composed of various materials, can affect bacterial contamination, adhesion, and subsequent biofilm formation. Biofilm formation by Pseudomonas aeruginosa is a major virulence factor. As catheterization duration increases, so does the risk of contamination and biofilm adhesion. The aim of this study was to investigate bacterial adherence to different Foley urinary catheter materials.

**Methods:**

A total of 300 used urinary catheters were analyzed for bacterial contamination. The bacteria isolated from these catheters were studied for biofilm formation using the tissue culture plate method. Sections of new Foley catheters made of latex, polyvinyl chloride (PVC), and silicone were exposed to a high biofilm-forming strain of P. aeruginosa to determine which material was more susceptible to bacterial attachment and biofilm formation. The surface morphology of the catheter materials was analyzed using scanning electron microscopy (SEM).

**Results:**

Of the 300 urinary catheters, 270 were contaminated with various uropathogens. A latex Foley catheter became contaminated with P. aeruginosa at 72 hours, while PVC and silicone catheters were infected at 120 hours. SEM analysis revealed biofilm formation on the catheter surfaces.

**Conclusions:**

Among the contaminated catheters, 49% contained P. aeruginosa, forming a consistent biofilm. Latex catheters were more susceptible to early infection compared to PVC and silicone.

## Introduction

Foley catheters have been in use since the 1930s as a primary method for managing urinary incontinence (1, 2). They consist of a flexible tube with an integrated balloon, typically made from materials such as polyvinyl chloride (PVC), latex rubber, polyurethane, and silicone (3). Natural rubber latex (NRL) is frequently used due to its remarkable wet gel strength, film-forming properties, elasticity, and flexibility (4). The attachment of bacteria to these materials and the subsequent formation of biofilms is a significant virulent factor in device-related infections. The ability of bacteria to adhere to the surface of biomaterials is influenced by the surface properties of both the material and the bacteria. Biofilm formation on catheters begins within minutes of catheterization (5) and plays a critical role in the development of catheter-associated urinary tract infections (CAUTIs) (6). CAUTIs account for 20% to 49% of all nosocomial infections, with approximately 90% of these infections associated with urinary catheter use (7).

In hospital settings, *Pseudomonas aeruginosa* is responsible for approximately 7-10% of urinary tract infections (8) and forms biofilms on urinary catheters through mechanisms such as alginate production, quorum sensing, and surface hydrophobicity modulation (9, 10). These biofilms are crucial to the pathogenicity of *P. aeruginosa*, contributing to persistent or recurrent infections (11). Prolonged catheterization increases the risk of infections and biofilm formation, leading to complications such as fever, bacteremia, urinary tract stones, obstruction, chronic pyelonephritis, chronic interstitial nephritis, renal failure, and bladder cancer (12-15). Bacterial attachment and biofilm formation can also cause catheter blockage, leading to frequent replacements, which increases costs, biomedical waste, and patient distress. Using materials that resist bacterial attachment could help reduce catheter-related infections.

This study aimed to investigate bacterial adhesion by *P. aeruginosa* on different urinary catheter materials. We examined latex, PVC, and silicone catheters to identify which material resists bacterial attachment and biofilm formation.

## Materials and Methods

**Screening of *P. aeruginosa* from used urinary catheters:** A total of 300 used urinary catheters were collected from patients in sterile containers and cut into equal segments. The segments were rinsed with sterile deionized water and transferred into sterile test tubes containing 10 mL of quarterstrength Ringer's solution (HiMedia Pvt. Ltd., Mumbai). Biofilms were disrupted by sonication for 5 minutes at 35 kHz, followed by vortex mixing for 2 minutes. The solution was cultured on Hi-chrome-UTI agar (HiMedia Pvt. Ltd., Mumbai), and bacterial colonies were identified after 24 hours of incubation.

**Biofilm formation by the isolates**: Biofilm formation by bacterial isolates was assessed using the tissue culture plate method. Bacteria were inoculated into trypticase soy broth containing 1% glucose and incubated for 24 hours at 37°C. The cultures were diluted 1:100 and 200 µL of the diluted cultures were added to each well of a 96-well polystyrene tissue culture plate. The plate was incubated for 24 hours at 37°C, followed by gentle tapping and washing to remove free-floating bacteria. The biofilm was fixed with 2% sodium acetate, stained with 0.1% crystal violet, and dried. The optical density (OD) at 570 nm was measured to determine the biofilm's strength. OD values greater than 0.240 were classified as strong biofilm-forming, 0.120-0.240 as moderate, and less than 0.120 as weak biofilm-forming (16).

**Evaluation of bacterial adherence on catheter materials**: Catheters made of latex, PVC, and silicone were cut into 1-2 cm sections and submerged in synthetic urine inoculated with a 0.5 Mac-Farland suspension of *P. aeruginosa*. The catheter sections were incubated for 24 hours, and one segment was removed at 24-hour intervals. The segments underwent washing with distilled water, phosphate-buffered saline, and normal saline to remove planktonic bacteria. Biofilms were then disrupted by vortexing, and the suspension was cultured on Hi-chrome UTI agar (HiMedia Pvt. Ltd., Mumbai) at 37°C for 24 hours.

**Surface morphology by environmental scanning electron microscopy (ESEM)**: Catheter sections were fixed in 2.5% glutaraldehyde, washed with sodium phosphate buffer, and subjected to ethanol washes at concentrations of 30%, 50%, and 80%. After air drying, the catheter sections were sent to IIT Powai for ESEM analysis using a FEI QUANTA 200 model, with a 20 µm scale bar. The magnification ranged from 25X to 20,000X, and images were captured in high vacuum, low vacuum, and ESEM modes.

## Results

**Figure 1:**
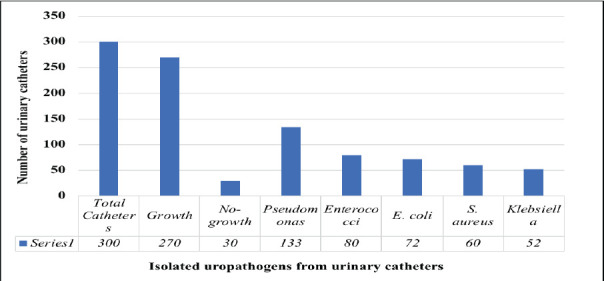
Uropathogens isolated from the urinary catheter

Among the 300 urinary catheters collected from hospitals in Amravati, 270 were contaminated with various uropathogens. Of these, 133 (49%) contained *P. aeruginosa*, the highest number among the isolates. Other organisms included Enterococci (80 catheters, 30%), *E. coli* (72 catheters, 27%), *Staphylococcus aureus* (60 catheters, 22%), and *Klebsiella* (52 catheters, 19%) (Figure 1).

**Figure 2:**
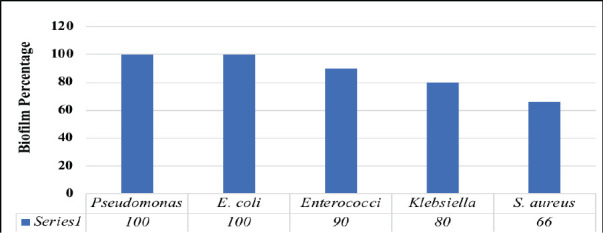
Percentage of biofilm formation by the isolates

**Biofilm development by isolates**: All *P. aeruginosa* and *E. coli* isolates exhibited 100% biofilm formation. Enterococci formed biofilms in 90% of cases, while *Klebsiella* and *Staphylococcus aureus* formed biofilms in 80% and 66% of cases, respectively (Figure 2).

**Bacterial adherence on catheter materials**: Bacterial growth was not observed on catheter pieces during the first 48 hours. However, latex material became infected with strong biofilm formation by *P. aeruginosa* at 72 hours, followed by PVC and silicone at 120 hours. From 96 hours onwards, microbial adherence was recorded on all three catheter materials. Control catheters exposed to synthetic urine without bacterial inoculation showed no bacterial growth.

**Figure 3:**
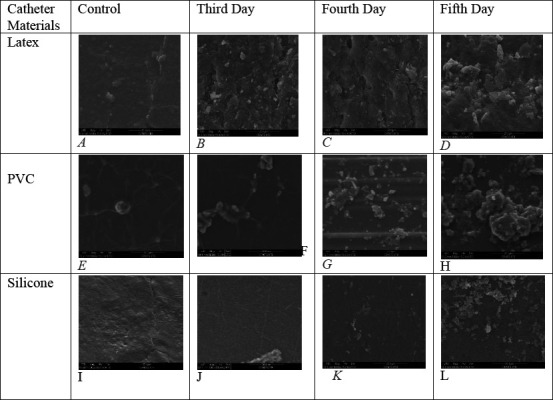
SEM images of different catheter materials showing biofilm formation by Pseudomonas aeruginosa (ESEM make: FEI, model: Quanta 200, scale bar, 20 µm) A, E, I: Control catheter surfaces with no bacterial attachment B, F, J: Catheter surfaces on 3^rd^ day of exposure to bacterial culture showing adherence on latex and PVC surfaces C, G, K: Catheter surfaces on 4^th^ day of exposure to bacterial culture showing biofilm formation on latex and PVC surfaces D, H, L: Catheter surfaces on 5^th^ day of exposure to bacterial culture showing dense biofilm on latex and PVC surfaces

**Surface morphology by ESEM**: ESEM images revealed bacterial biofilm on all three types of catheter materials, with biofilm density increasing as exposure duration increased. The control catheters did not show bacterial attachment. SEM images confirmed biofilm formation on latex and PVC surfaces after 72 hours of exposure, with dense biofilm observed on latex and PVC by day 5 (Figure 3).

## Discussion

This study aimed to evaluate bacterial adhesion and biofilm formation by *P. aeruginosa* on different Foley catheter materials. *P. aeruginosa*, a biofilmforming bacterium, was isolated from contaminated catheters and allowed to adhere to latex, PVC, and silicone catheters. The findings highlight the importance of selecting materials that resist bacterial colonization to reduce catheter-related infections.

Among the 300 catheters, 270 were contaminated with various uropathogens, with *P. aeruginosa* being the most prevalent. All isolates were significant biofilm producers, with 100% biofilm formation observed in *P. aeruginosa* and *E. coli* isolates. This result aligns with previous studies showing that *P. aeruginosa* is a major contributor to nosocomial infections (18, 19).

Our study revealed that latex catheters were more susceptible to early infection compared to PVC and silicone catheters. By 72 hours, latex catheters showed strong biofilm formation, whereas PVC and silicone catheters required 120 hours to become infected. These findings support the idea that silicone catheters, due to their biocompatibility and resistance to bacterial adhesion, may reduce the risk of CAUTIs and improve patient comfort.

Additionally, the SEM analysis demonstrated that silicone catheters resisted bacterial attachment for the longest duration, consistent with findings from previous studies (27). However, while silicone catheters reduce bacterial adherence, they do not completely eliminate biofilm formation, emphasizing the need for continued research into more effective materials and designs.

In conclusion, the study found that *P. aeruginosa* was the most common pathogen isolated from contaminated catheters, and it formed biofilms on all catheter materials. Latex catheters became infected earlier than PVC and silicone, with silicone providing the longest resistance to bacterial attachment. Future research should focus on developing new biomaterials and catheter designs to further reduce bacterial colonization and biofilm formation, thereby preventing catheter-associated urinary tract infections.
